# Exposure–response analysis of pertuzumab in HER2-positive metastatic breast cancer: absence of effect on QTc prolongation and other ECG parameters

**DOI:** 10.1007/s00280-013-2279-6

**Published:** 2013-09-03

**Authors:** Amit Garg, Jing Li, Emma Clark, Adam Knott, Timothy J. Carrothers, Jean-François Marier, Javier Cortés, Michael Brewster, Jennifer Visich, Bert Lum

**Affiliations:** 1 Genentech, Inc., 1 DNA Way, MS-463A, South San Francisco, CA 94080 USA; 2 F. Hoffmann-La Roche Ltd, 6 Falcon Way, Shire Park, Hexagon Place, Welwyn Garden City, Hertfordshire AL7 1TW UK; 3 Pharsight, Inc., 100 Mathilda Place, Suite 160, Sunnyvale, CA 94086 USA; 4 Vall d’Hebron University Hospital, Vall d’Hebron Institute of Oncology (VHIO), Passeig Vall d’Hebron 119, Edifici Maternoinfantil Planta 14, 08035 Barcelona, Spain; 5 Present Address: MedImmune, 24500 Clawiter Road, Hayward, CA 94545 USA; 6 Present Address: Forest Research Institute/Cerexa, Inc., 2100 Franklin Street, Suite 900, Oakland, CA 94612 USA

**Keywords:** Cardiac repolarization, HER2-positive metastatic breast cancer, Pertuzumab, QT

## Abstract

**Purpose:**

The phase III trial of pertuzumab plus trastuzumab plus docetaxel versus placebo plus trastuzumab plus docetaxel for first-line treatment of HER2-positive metastatic breast cancer included a substudy to determine whether pertuzumab affected the corrected QT (QTc) interval or other electrocardiogram parameters.

**Methods:**

Triplicate 12-lead electrocardiogram measurements and serum samples were collected before (–30 and –15 min) and after (0–15 and 60–75 min) pertuzumab/placebo infusions (Cycles 1 and 3), and at 72 h post-infusion (Cycle 1). Fridericia’s correction was applied to QT measurements (QTcF) and change from baseline (ΔQTcF) calculated. Statistical analyses were performed on baseline-adjusted, placebo-corrected QTcF values (ΔΔQTcF). Linear mixed-effects modeling evaluated potential exposure–response relationships between ΔQTcF and observed pertuzumab concentrations.

**Results:**

Thirty-seven female patients participated in the substudy. QTcF values in both groups were within the normal range and below critical thresholds of clinical concern. No pertuzumab-treated patient showed abnormal electrocardiogram morphology. In Cycle 1, mean ΔΔQTcF (90 % CI) values at 0–15 min, 60–75 min, and 72 h post-infusion were −6.96 (−13.69, −0.23), −6.35 (−13.57, 0.88), and −4.08 (−12.64, 4.48), all of which were <5 ms, with upper CI limits <10 ms. One Cycle 3 post-infusion mean ΔΔQTcF value exceeded 5 ms. Other electrocardiogram parameters were within normal ranges. Concentration–QTc modeling showed no apparent relationship between ΔQTcF and pertuzumab concentrations.

**Conclusions:**

Cardiac monitoring and concentration–QTc modeling demonstrated that pertuzumab, combined with trastuzumab and docetaxel, had no clinically relevant effects on QTcF and other electrocardiogram parameters.

**Electronic supplementary material:**

The online version of this article (doi:10.1007/s00280-013-2279-6) contains supplementary material, which is available to authorized users.

## Introduction

Although improved early detection and advances in systemic therapy for early stage disease have resulted in a decline in breast cancer mortality in recent years [1, 2], metastatic breast cancer (MBC) remains essentially incurable. Human epidermal growth factor receptor 2 (HER2), a cell-surface receptor involved in regulation of cell growth, survival, and differentiation [3], has emerged as one of the most important targets in breast cancer treatment. Around 15–20 % of breast cancers exhibit amplification and/or overexpression of HER2 (“HER2-positivity”) [4–6], which is associated with increased tumor aggressiveness, higher rates of recurrence, and increased mortality [6–11]. There is a significant need for new anti-HER2 agents with novel mechanisms of action and non-overlapping toxicity, which can be combined with established treatments for breast cancer.

Pertuzumab (rhuMAb 2C4) is a humanized monoclonal anti-HER2 antibody that prevents heterodimerization of HER2 with other members of the HER family (HER1, HER3, and HER4), thus inhibiting ligand-activated downstream signaling [12]. The combination of pertuzumab, with trastuzumab, another HER2-targeted humanized monoclonal antibody, and docetaxel is indicated for first-line treatment of HER2-positive MBC [13]. Although both antibodies target HER2, pertuzumab and trastuzumab bind to distinct epitopes in the extracellular domain (ECD) of the receptor and have complementary mechanisms of action [14]. While pertuzumab prevents the ligand-activated formation of HER2 heterodimers, trastuzumab prevents the shedding of the HER2 ECD (thereby blocking formation of constitutively active truncated receptors) and disrupts ligand-independent HER2–HER3–phosphatidylinositol 3-kinase (PI3 K) complex formation [14–16].

The efficacy and safety of pertuzumab, in combination with trastuzumab plus docetaxel for the first-line treatment of HER2-positive MBC, were demonstrated in the international, randomized, double-blind, placebo-controlled phase III CLEOPATRA trial, which involved approximately 800 patients [13, 17]. In this study, pertuzumab was administered every 3 weeks by IV infusion at an initial dose of 840 mg in Cycle 1, followed by 420 mg in subsequent cycles. The results of the primary endpoint demonstrated a significant increase in progression-free survival (PFS) with pertuzumab plus trastuzumab plus docetaxel, as compared with placebo plus trastuzumab plus docetaxel, with a 6.1-month increase in median PFS with pertuzumab-containing therapy [13, 17]. Overall survival was also significantly improved in the pertuzumab arm compared with the control arm [18].

Novel pharmaceutical agents should undergo rigorous evaluation for their potential to delay cardiac repolarization [19]. Assessed as prolongation of the QT interval on the electrocardiogram (ECG), a delay in cardiac repolarization creates an electrophysiological environment that favours the development of ventricular arrhythmias, most notably torsade de pointes (TdP), which may lead to sudden death. The International Conference on Harmonisation (ICH) E14 document recommends that all systemically available drugs, other than those intended for the control of arrhythmias, should undergo careful clinical testing in a thorough QT/corrected QT (QTc) interval study [19, 20]. However, challenges exist in implementing a thorough QT study for oncology drugs; for example, potential toxicities preclude administration of therapeutic doses to healthy volunteers or supratherapeutic doses to patients with cancer, and prolonging periods without active treatment is unethical [21]. Moreover, monoclonal antibodies such as pertuzumab are expected to have a lower potential to affect the QT interval due to their large molecular size, which precludes direct access to the hERG channel drug-binding site, and high target specificity compared with small-molecule agents [22]. Of note, trastuzumab, another anti-HER2 antibody, was found to have no significant effect on the QT interval or other ECG parameters when administered to patients with MBC [23], whereas docetaxel has been associated with a proarrhythmogenic effect [24].

In cases where a thorough QT study in healthy volunteers is considered impractical or unethical, dedicated ECG monitoring for evaluation of possible effects on the QTc interval, PR interval, and heart rate (HR), supported by concentration–QTc modeling, can be considered as an alternative approach to investigate potential drug-induced cardiac effects [19, 20]. Therefore, a substudy of the phase III CLEOPATRA trial was conducted to determine whether pertuzumab affected cardiac repolarization when combined with trastuzumab and docetaxel for the first-line treatment of MBC. ECG and pharmacokinetic (PK) data were collected during Cycles 1 and 3 in order to characterize the QTc interval and other ECG parameters during study treatment, and to assess potential concentration–QTc relationships.

## Methods

### Substudy design and ethics

CLEOPATRA was a phase III trial performed to assess the efficacy and safety of pertuzumab (PERJETA^®^, F. Hoffmann-La Roche, Basel, Switzerland; Genentech Inc., South San Francisco, CA) plus trastuzumab plus docetaxel, as compared with placebo plus trastuzumab plus docetaxel, as first-line treatment for patients with HER2-positive MBC [13, 17]. The trial was conducted in full accordance with the guidelines for Good Clinical Practice and the Declaration of Helsinki. A subset of clinical sites from the CLEOPATRA study participated in the PK/QTc substudy with the goal of assessing the effect of pertuzumab on cardiac repolarization. The protocol of the substudy, the Informed Consent Form, and relevant supporting information were submitted to an Institutional Review Board (IRB) or Ethics Committee (EC) for review and approval before initiation of the substudy. The Principal Investigator was responsible for providing written summaries of the status of the study to the IRB/EC. Written informed consent was obtained from each individual participating in this study, after adequate explanation of the aims, methods, anticipated benefits, objectives, and potential hazards.

### Patients and treatment

Patients enrolled in the substudy received the same treatments as specified in the main CLEOPATRA study. In the control arm, patients received a pertuzumab placebo infusion starting on Day 1 of Cycle 1, plus a trastuzumab 8 mg/kg IV loading dose (followed by 6 mg/kg IV every 3 weeks until disease progression or unacceptable toxicity) and docetaxel 75 mg/m^2^ IV (every 3 weeks for at least six cycles, or until disease progression or unacceptable toxicity) starting on Day 2 of Cycle 1. Treatment in the experimental arm included a pertuzumab 840 mg IV loading dose starting on Day 1 of Cycle 1 (followed by 420 mg IV every 3 weeks until progressive disease or unacceptable toxicity), plus trastuzumab and docetaxel administered as in the control arm. Study treatment cycles were 3 weeks (21 days) in length.

The first dose of pertuzumab/placebo (Cycle 1, Day 1) was planned within 3 days of randomization. The first dose of trastuzumab was administered 24 h later (Cycle 1, Day 2), followed by the first dose of docetaxel on the same day. If the initial infusions of all three agents were well tolerated, as determined by the investigator, subsequent doses of trastuzumab and docetaxel could also be administered on Day 1 of each cycle. At the discretion of the treating physician, the docetaxel dose could be increased to 100 mg/m^2^ according to tolerability. The following treatment sequence was used when all drugs were given on the same day: pertuzumab/placebo, trastuzumab, and docetaxel.

#### ECG and PK data collection

Twelve-lead ECG measurements were obtained in triplicate from resting, supine patients before (−30 and −15 min) and after (0–15 and 60–75 min) pertuzumab/placebo infusion on Day 1 of Cycles 1 and 3, and on Day 3 of Cycle 1 (approximately 72 h after the pertuzumab/placebo infusion). Blood draws and other procedures were avoided immediately before ECG data collection, and timing of meals was standardized as much as possible between patients. ELI 250 (Mortara Instrument, Inc., Milwaukee, WI) machines were supplied to substudy sites and used with standard lead placement. The same machine was used for all ECGs obtained from each individual patient. Raw ECG data were sent to a central core cardiology laboratory, where ECG readers, who were blinded to treatment and ECG time point, produced a single dataset for automated analysis. ECG measurements included QRS duration, PR interval, HR, QT intervals, RR intervals, U waves, T waves, and instances of abnormal ECG morphology.

Blood samples were drawn immediately after the corresponding ECG assessments for PK analyses. Serum pertuzumab concentrations were measured with a validated bridging enzyme-linked immunosorbent assay (ELISA), which used a monoclonal anti-idiotype antibody to capture pertuzumab from serum samples. The minimum quantifiable concentration of pertuzumab in serum was 150 ng/ml [25].

### Statistical analyses

Demographic data and baseline characteristics were summarized with descriptive statistics for the two treatment groups. In order to reduce the dependence of QT on HR, Fridericia’s correction was applied (QTcF = QT/RR^0.33^) [19]. Bazett’s formula was additionally used to correct for HR, but was found to provide poorer correction compared with Fridericia’s method (data not shown). All presented analyses are therefore based on QTcF.

Individual QTcF measurements were summarized with descriptive statistics by cycle, treatment, and time point. Incidences of abnormal ECG results of clinical and regulatory interest [19] at screening and post-screening were tabulated and summarized using graphical displays. These included: new incidences of QTcF values >450, >480, or >500 ms; change from baseline in QTcF (ΔQTcF) >30 or >60 ms; change from baseline HR (ΔHR) ≥25 %, resulting in final HR <50 or >120 bpm; change from baseline PR (ΔPR) ≥25 %, resulting in final PR >200 ms; change from baseline QRS (ΔQRS) ≥25 %, resulting in final QRS >110 ms; and new incidences of abnormal U waves, T waves, or ECG morphology.

Baseline ECG was defined as the average of pre-dose observations at Cycle 1, Day 1 (i.e., 15 min and 30 min prior to infusion), and this Cycle 1 baseline was used for all analyses in the substudy (including those in Cycle 3). Baseline-adjusted, placebo-corrected QTcF (ΔΔQTcF) values were derived using the following formula:

$$\Updelta \Updelta {\text{QTcF}} = \left( {{\text{mean of }}\Updelta {\text{QTcF for pertuzumab group}}} \right)-\left( {{\text{mean of }}\Updelta {\text{QTcF for placebo group}}} \right).$$

Descriptive statistics of ΔΔQTcF were presented by treatment, cycle, and time point. Point estimates of ΔΔQTcF and two-sided 90 % confidence intervals (CIs) were derived by inverting the results of a *t* test. The variance of the difference of means was calculated using either a pooled or Satterthwaite estimate of the variance depending on the *p* value of the *F* test for equality of variances (*α* = 0.10). Descriptive and inferential statistics were calculated using SAS Version 9.2 (SAS Institute Inc., Cary, NC).

The concentration–QTcF relationship was explored using linear mixed-effects analyses [26]. The dataset consisted of observed drug concentrations and ΔQTcF values collected on Day 1 of Cycles 1 and 3. For patients who received placebo group treatment, concentrations were set to zero. Data points were excluded if either the ECG or concentration data were missing. The concentration–QTcF relationship was assessed according to the following equation [26]:

$$Y = \alpha + \beta * \, \left[ {\text{pertuzumab}} \right] \, + \varepsilon ,$$

where *Y* is the response variable (i.e., ΔQTcF), the intercept *α* represents the mean response, and the slope *β* represents the change in mean for a unit change in pertuzumab serum concentration. The statistical significance of the slope parameter (*β*) corresponds to the following hypothesis testing:

$${\text{H}}_{0} :\beta = \, 0 \quad {\text{ and \quad H}}_{ 1} :\beta \ne \, 0.$$

Using a statistical criterion of *p* < 0.05, this corresponds to a change in the objective function, defined as (−2) * log-likelihood, of 3.83 units. Interindividual variability, as a random effect (an additive term), was estimated for intercept (*α*) and, if possible, slope (*β*), as well as their correlation. Random effects were assumed to be normally distributed with mean zero and variance *ω* ^2^. The matrix Ω becomes diagonal when the correlation is zero. The additive measurement error *ε* was assumed to be normally distributed with mean zero and unknown constant variance *σ* ^2^ [26]. Graphical presentation and linear mixed-effects modeling were performed using TIBCO Spotfire S-Plus^®^ software, Version 8.1 (TIBCO Spotfire Inc., Somerville, MA).

## Results

### Patient demographics

Descriptive statistics of demographic data and other baseline characteristics in patients from the substudy were similar between the two arms and were consistent with those of the overall CLEOPATRA study population (Supplementary Table 1) [13]. In total, 37 female patients were enrolled in the substudy, of whom 20 received pertuzumab plus trastuzumab plus docetaxel and 17 received placebo plus trastuzumab plus docetaxel. The mean age was 53.1 years, and a total of 33 patients (89.2 %) were <65 years of age. Substudy participants had a mean weight of 70.9 kg.

### QTcF

Descriptive statistics of QTcF data by cycle, treatment, and time point are presented in Table 1. Of note, mean baseline QTcF, defined as the mean of the raw QTcF values at both pre-infusion time points in Cycle 1, was 410.7 ms in the pertuzumab group and 420.0 ms in the placebo group. In Cycle 1, mean and median QTcF pre-infusion time point values were consistent with values at the 0–15 min and 60–75 min post-infusion time points for both treatment groups. Similarly, pre-infusion mean and median QTcF values in Cycle 3 were consistent with those observed post-infusion for the pertuzumab and placebo groups. Absolute QTcF values were within the normal range for women and below critical thresholds associated with the development of TdP/sudden death [27]. In the placebo group, mean QTcF on Day 3 of Cycle 1 (420.5 ms) was similar to values observed on Day 1 at 0–15 min and 60–75 min post-infusion (420.5 and 419.4 ms, respectively); suggesting that docetaxel treatment on Day 2 had no effect on QTcF on Day 3.

**Table 1:** QTcF in Cycles 1 and 3, by treatment arm

Time point		QTcF (ms)					
		Placebo + trastuzumab + docetaxel			Pertuzumab + trastuzumab + docetaxel		
		*n*	Mean ± SD	Median (range)	*n*	Mean ± SD	Median (range)
Cycle 1	30 min prior to infusion	15	420.5 ± 21.77	425.7 (375.0, 466.5)	18	411.3 ± 15.10	417.7 (378.3, 432.7)
	15 min prior to infusion	15	419.4 ± 20.40	424.3 (367.7, 444.0)	18	410.1 ± 17.29	416.7 (374.7, 433.0)
	0–15 min post-infusion	15	426.9 ± 19.19	425.3 (391.7, 451.0)	20	415.9 ± 18.35	419.5 (367.3, 444.3)
	60–75 min post-infusion	17	426.6 ± 18.13	423.3 (380.0, 451.0)	17	410.5 ± 18.98	414.0 (374.7, 431.3)
	72 h post-infusion	17	420.5 ± 11.06	418.7 (394.3, 439.0)	17	409 ± 13.80	409.7 (386.0, 431.3)
Cycle 3	30 min prior to infusion	17	411.9 ± 19.01	413.7 (374.7, 440.7)	19	407.6 ± 17.10	408.0 (362.3, 431.0)
	15 min prior to infusion	17	410.1 ± 17.47	411.0 (378.3, 452.0)	19	405.8 ± 17.53	408.0 (354.7, 430.0)
	0–15 min post-infusion	17	415.2 ± 21.77	416.7 (379.0, 451.7)	19	413.2 ± 16.23	416.7 (374.3, 438.3)
	60–75 min post-infusion	17	416.1 ± 21.49	415.3 (375.0, 453.3)	19	407.9 ± 18.25	410.7 (369.7, 436.7)

*QTcF* QT interval, corrected for heart rate using Fridericia’s correction

**Fig. 1:**
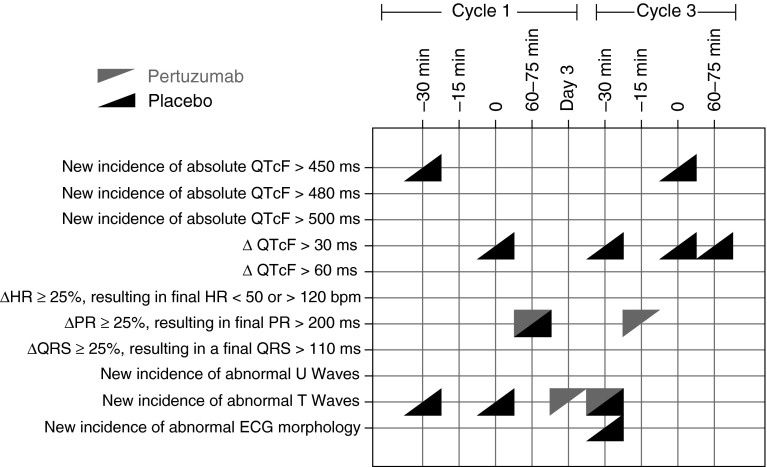
Summary of incidence of ECG abnormalities by cycle and time point.* Triangles* indicate that at least one pertuzumab-treated patient (*gray triangles*) or placebo-treated patients (*black triangles*) had a positive test result at that time point. *ECG* electrocardiogram, *QTcF* QT interval, corrected for heart rate using Fridericia’s correction

Abnormal ECG results of clinical and regulatory interest were analyzed for both treatment groups (Fig. 1). Overall, no patient in the pertuzumab arm showed QTcF values of >450 ms, whereas two patients in the placebo arm had QTcF values of >450 ms; however, there were no incidences of QTcF values of >480 ms or >500 ms in either treatment group. No changes from baseline in QTcF of >30 ms occurred in the pertuzumab group, whereas such changes were recorded for four patients in the placebo group. Changes from baseline in QTcF did not exceed 60 ms for any patient enrolled in the substudy.

### ΔQTcF and ΔΔQTcF

To further assess the potential effect of study treatment in the pertuzumab arm relative to that in the placebo arm, summary statistics of ΔQTcF and ΔΔQTcF in Cycles 1 and 3 were prepared (Table 2; Supplementary Fig. 1). In Cycle 1, upper ranges of ΔQTcF for the pertuzumab group were <30 ms for all three post-infusion time points. Point estimates of ΔΔQTcF measured 0–15 min, 60–75 min, and 72 h post-infusion were −6.96, −6.35, and −4.08 ms, respectively, all of which were <5 ms, with upper limits of the corresponding 90 % CIs of <10 ms.

**Table 2:** ΔQTcF in Cycles 1 and 3 by treatment arm, and resulting ΔΔQTcF

Cycle	Time point post-infusion	ΔΔQTcF (ms)						ΔΔQTcF (ms), Mean (90 % CI)
		Placebo + trastuzumab + docetaxel			Pertuzumab + trastuzumab + docetaxel			
		*n*	Mean ± SD	Median (range)	*n*	Mean ± SD	Median (range)	
Cycle 1	0–15 min	15	9.32 ± 12.99	12 (−21.92, 34.83)	18	2.36 ± 9.81	2.92 (−16.67, 20.17)	−6.96 (−13.69, −0.23)
	60–75 min	15	6.69 ± 10.87	8.67 (−20.58, 18.83)	17	0.34 ± 12.93	−2.17 (−16.00, 29.83)	−6.35 (−13.57, 0.88)
	72 h	15	0.54 ± 15.69	−1 (−25.58, 29.50)	17	−3.54 ± 12.83	−2.83 (−26.83, 16.33)	−4.08 (−12.64, 4.48)
Cycle 3	0–15 min	15	−6.39 ± 21.5	−5.92 (–38.67, 44.67)	17	2.02 ± 13.17	−1.0 (−15.17, 23.33)	8.41 (−2.58, 19.39)
	60–75 min	15	−4.41 ± 21.55	−6.92 (−38.00, 46.33)	17	–4.45 ± 15.19	−7.5 (−28.83, 25.83)	−0.04 (−11.12, 11.04)

*CI* confidence interval, *QTcF*, QT interval, corrected for heart rate using Fridericia’s correction, *ΔQTcF*, baseline-adjusted QTcF, *ΔΔQTcF* baseline-adjusted, placebo-corrected QTcF, *SD* standard deviation

In Cycle 3, mean ΔQTcF values for both post-infusion time points in the pertuzumab and placebo groups were <5 ms. Variability of ΔQTcF data in the placebo group was markedly higher than that observed in the pertuzumab group. Mean values of ΔΔQTcF for the 0–15 min and 60–75 min post-infusion time points were 8.41 ms (90 % CI −2.58, 19.39) and −0.04 ms (90 % CI −11.12, 11.04), respectively. Although the upper limits of the 90 % CIs for both time points were >10 ms, the 90 % CIs also included 0 ms. Importantly, the Cycle 3 post-infusion QTcF values in the placebo arm were lower than baseline (i.e., pre-infusion Cycle 1), leading to lower point estimates of ΔQTcF in the placebo arm in Cycle 3. The resulting overcorrection would then account for the inflation of ΔΔQTcF estimates, rather than a true drug effect on QTcF.

### Concentration–QTcF modeling

The dataset for the exposure–response analysis contained 33 patients with baseline QTc data and at least one subsequent QTc observation with a corresponding PK sample. In the pertuzumab group, mean (± standard deviation) serum pertuzumab concentrations were 272 ± 49 μg/ml at 60–75 min post-infusion in Cycle 1, 65 ± 49 μg/ml at 15 min pre-infusion in Cycle 3, and 186 ± 33 μg/ml at 60–75 min post-infusion in Cycle 3. Pertuzumab arm of all patients had measureable serum pertuzumab concentrations prior to the Cycle 3 infusion (range 19–245 μg/ml).

**Fig. 2:**
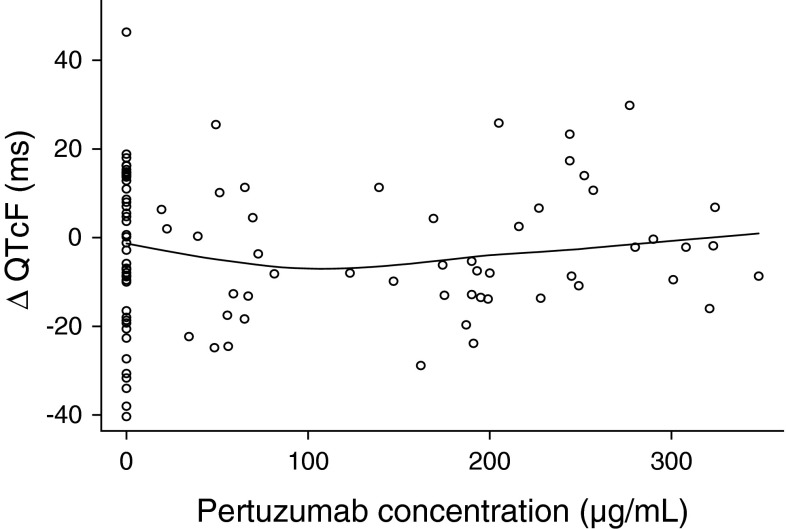
Plot of serum pertuzumab concentrations versus ΔQTcF in Cycles 1 and 3. The *black line* is a LOESS smooth curve with 70 % span.* QTcF* QT interval, corrected for heart rate using Fridericia’s correction

**Fig. 3:**
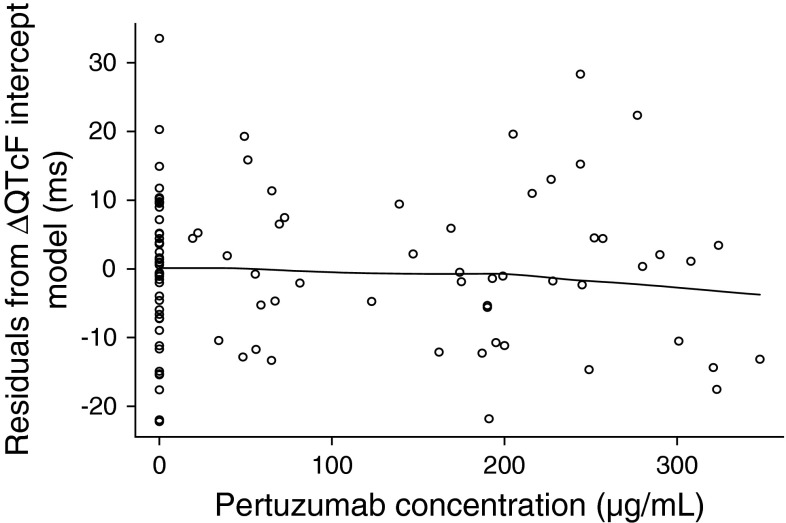
Plot of residuals of predicted ΔQTcF versus observed serum pertuzumab concentrations of pertuzumab. The *black line* is a LOESS smooth curve with 70 % span. *QTcF* QT interval, corrected for heart rate using Fridericia’s correction

An exploratory analysis was performed to assess the shape of the concentration–QTcF relationship. As shown in Fig. 2, there was no apparent relationship between individual serum pertuzumab concentrations and ΔQTcF in Cycles 1 and 3. Because the exploratory data analysis identified intercycle variability in intercept (α) between Cycles 1 and 3, a cycle-specific intercept was tested for statistical significance. Results of the linear mixed-effects model building are presented in Table 3. The slope estimate of −0.0093 with standard error (SE) of 0.0167 was not statistically significant (*p* > 0.05), indicating no apparent relationship between ΔQTcF and pertuzumab serum concentrations. A statistically significant difference in intercept by cycle was observed, with a mean (±SE) difference of −9.5 ± 2.8 ms between Cycles 3 and 1, as a result of intercycle variability in baseline QTcF. Residual intra-patient variability (the standard deviation of ΔQTcF within a patient) was 12.3 ms, expressed as the square root of the estimated variance. Residuals of ΔQTcF derived from the final model were homogeneously distributed around 0, suggesting no bias in predicting high and low values of ΔQTcF (Fig. 3).

**Table 3:** Parameter estimates and variability derived with final concentration QTcF linear mixed-effects model

Parameter	Estimate	SE	CV %	95 % CI (SE-derived)
Intercept for Cycle 1 (ms)	3.4	2.8	82 %	−2.1, 8.9
Between-subject variability (ms)	9.4			
Difference in intercept (Cycle 3 vs. Cycle 1)	−9.5	2.8	29 %	−15.0, −4.0
Slope (ms/μg/ml)	NS	–	–	
Residual variability (ms)	12.3			

*CI* confidence interval, *CV* coefficient of variation, *QTcF* QT interval, corrected for heart rate using Fridericia’s correction, *ΔQTcF* baseline-adjusted QTcF, *ΔΔQTcF* baseline-adjusted, placebo-corrected QTcF, *SE* standard error

## Discussion

Prolongation of the QTc interval, resulting in increased susceptibility to cardiac arrhythmia, is a recognized property of many pharmaceutical agents used across a wide range of therapeutic settings. Novel, systemically available agents should ideally undergo a thorough QT evaluation in healthy volunteers, but where such a study is deemed impractical or unethical, dedicated ECG monitoring supported by concentration–QTc modeling is recommended to investigate potential drug-induced cardiac effects [19, 20]. Since pertuzumab is an anti-HER2 IgG1 monoclonal antibody with a long half-life of approximately 18 days [28], a multiple-dose study in healthy volunteers or a crossover study in HER2-positive MBC involving a washout period and placebo treatment was not deemed ethical. Therefore, a parallel-design study was performed in the target patient population treated with the intended marketed dose and schedule of pertuzumab (i.e., 840 mg IV loading dose followed by 420 mg IV every 3 weeks). This substudy was conducted in a subset of patients enrolled in the CLEOPATRA study to evaluate the effect of pertuzumab on cardiac repolarization when administered in combination with trastuzumab and docetaxel in patients with HER2-positive MBC.

In Cycle 1, the upper range of ∆QTcF for the pertuzumab group was <30 ms at all post-infusion time points, and point estimates of ΔΔQTcF were all <5 ms, with corresponding upper limits of the 90 % CIs <10 ms. These findings indicate that pertuzumab did not prolong QTcF in this first cycle, during which pertuzumab serum concentrations were at their highest owing to administration of the loading dose. In Cycle 3, mean ΔQTcF values for both post-infusion time points in the pertuzumab and placebo groups were <5 ms. Mean ΔΔQTcF for the 0–15 min and 60–75 min post-infusion time points in Cycle 3 was 8.41 and −0.04 ms, respectively, and 90 % CIs for both values included 0 and 10 ms. Importantly, mean baseline QTcF was 9.3 ms lower in the pertuzumab arm compared with the placebo arm (410.7 vs. 420.0 ms, respectively), and the Cycle 3 post-infusion QTcF values in the placebo arm were lower than baseline leading to lower point estimates of ΔQTcF in the placebo arm in Cycle 3. As a result, ΔΔQTcF values may have been inflated due to the overcorrection associated with the low ΔQTcF in the placebo group.

In assessing the findings of this substudy, it is important to consider two additional factors: the normal QTc and the absolute value of clinical concern. The E14 guidance document notes that QTc prolongation of >500 ms or QTc interval increases from baseline of >60 ms are commonly used thresholds at which drug discontinuation may be considered in a given individual [19, 20, 29]. In the present substudy, no pertuzumab-treated patients had absolute QTcF values of >450 ms or ΔQTcF of >30 ms, further supporting a lack of clinically meaningful effect of pertuzumab on the QT interval when combined with trastuzumab and docetaxel.

Additional evidence for the absence of a QT effect was provided by the exposure–response model, which showed no apparent relationship between serum pertuzumab concentrations and ΔQTcF. There are limitations to using a concentration–QTc modeling approach to investigate potential cardiac effects of monoclonal antibodies targeting cell-surface proteins, since such agents often exhibit nonlinear pharmacokinetics and serum exposures may not directly correlate with pharmacodynamic effects [22]. However, pertuzumab shows linear pharmacokinetics at clinical doses [28], and point estimates of ΔQTcF were generally unremarkable irrespective of the time point analyzed.

The absence of clinically relevant effect of pertuzumab on the QTc interval is consistent with its large molecular size, which precludes direct access to the hERG channel drug-binding site. Similar results have been reported from a small study of trastuzumab in 20 patients with HER2-positive MBC, in which no significant changes in ECG parameters, including QT and RR intervals or QT dispersion, were noted following drug infusion [23]. These clinical findings are also consistent with results from two multi-dose toxicology studies in cynomolgus monkeys. Following administration of pertuzumab at doses of up to 150 mg/kg for more than 26 weeks, there was no evidence of cardiac injury in either of these nonclinical studies, as evidenced by histopathology, lack of elevations in serum cardiac markers, and normal ECGs, blood pressures and heart rates (unpublished data on file, Genentech, Inc.). Although taxanes have been associated with a certain potential for cardiotoxicity [24], the present substudy found no evidence that docetaxel treatment, when initiated on Day 2 of Cycle 1, affected QTcF.

It is important to acknowledge the limitations of the present substudy compared with a thorough QT study. Of note, the omission of a positive control (e.g., moxifloxacin) meant that it was not possible to confirm the sensitivity of the assay. However, the design of the substudy reflects the practical and ethical constraints of the treatment setting and is consistent with the recommendations of the Cardiac Safety Research Consortium on QT assessment for therapeutic proteins, which are expected to have low potential to affect cardiac electrical activity [22].

In conclusion, statistical analyses of ΔQTcF and ΔΔQTcF and results of concentration–QTc modeling in the current substudy suggest that pertuzumab has no clinically relevant effect on QTcF and other ECG parameters in patients with HER2-positive MBC. Absolute QTcF values were within the normal range for women and below threshold values associated with signals of clinical relevance in the development of TdP/sudden death [27].

## Electronic supplementary material

Below is the link to the electronic supplementary material.

Supplementary material 1 (DOCX 15 kb)

Supplementary material 2 (DOCX 313 kb)
