# Supplementary material for: Exposure–response analysis of pertuzumab in HER2-positive metastatic breast cancer: absence of effect on QTc prolongation and other ECG parameters
Source: Cancer Chemother Pharmacol. 2013 Sep 3;72(5):1133–41. doi: 10.1007/s00280-013-2279-6 (PMC3825499; doi:10.1007/s00280-013-2279-6)
Supplement: Supplementary file 1 — Supplementary material 1 (DOCX 15 kb) [file 280_2013_2279_MOESM1_ESM.docx]

**Supplementary Table 1** Baseline characteristics

|  | Placebo + trastuzumab + docetaxel (*n* = 17) | Pertuzumab + trastuzumab + docetaxel (*n* = 20) |
| --- | --- | --- |
| Female, *n* (%) | 17 (100.0) | 20 (100.0) |
| Race, *n* (%) Asian White Other | 4 (23.5) 11 (64.7) 2 (11.8) | 4 (20.0) 14 (70.0) 2 (10.0) |
| Mean age, years (range) | 55.3 (36, 67) | 51.2 (22, 66) |
| Age group, *n* (%) <65 years ≥65 years | 14 (82.4)  3 (17.6) | 19 (95.0) 1 (5.0) |
| Mean weight, kg (range) | 71 (48, 98) | 70.9 (52, 98) |
| Region, *n* (%) Asia Europe North America | 3 (17.6) 11 (64.7) 3 (17.6) | 4 (20.0) 14 (70.0) 2 (10.0) |
| Female reproductive status, *n* (%) Postmenopausal Surgically sterilized Childbearing potential with contraceptive protection | 11 (64.7) 3 (17.6) 3 (17.6) | 15 (75.0) 2 (10.0) 3 (15.0) |
| Smoking status, *n* (%) Current smoker  Never smoked  Past smoker | 4 (23.5) 11 (64.7) 2 (11.8) | 2 (10.0) 16 (80.0) 2 (10.0) |
| Mean LVEF, % (range) | 64.4 (51, 88) | 66.9 (54, 78) |
| Normal chest X-ray, *n* (%) | 12 (70.6) | 13 (65.0) |
| ECOG status, *n* (%) 0 1 | 15 (88.2) 2 (11.8) | 14 (70.0) 6 (30.0) |
| Prior treatment status, *n* (%) (Neo)adjuvant *De novo* | 14 (82.4) 3 (17.6) | 13 (65.0) 7 (35.0) |
| Visceral disease, *n* (%) | 10 (58.8) | 18 (90.0) |
| Measurable disease^a^, *n* (%) | 11 (64.7) | 18 (90.0) |

^a^ As assessed by independent review facility. ECOG, Eastern Cooperative Oncology Group; LVEF, left ventricular ejection fraction
