# Supplementary material for: Exposure–response analysis of pertuzumab in HER2-positive metastatic breast cancer: absence of effect on QTc prolongation and other ECG parameters
Source: Cancer Chemother Pharmacol. 2013 Sep 3;72(5):1133–41. doi: 10.1007/s00280-013-2279-6 (PMC3825499; doi:10.1007/s00280-013-2279-6)
Supplement: Supplementary file 2 — Supplementary material 2 (DOCX 313 kb) [file 280_2013_2279_MOESM2_ESM.docx]

**Supplementary Fig. 1** Mean ΔΔQTcF (solid line) and 90% CI (dotted lines) in Cycles 1 and 3. CI, confidence interval; ΔΔQTcF, baseline-adjusted, placebo-corrected QT interval, corrected for heart rate using Fridericia’s correction
